# Identification of a tripartite interaction between the N-terminus of HIV-1 Vif and CBFβ that is critical for Vif function

**DOI:** 10.1186/s12977-017-0346-5

**Published:** 2017-03-17

**Authors:** Belete A. Desimmie, Jessica L. Smith, Hiroshi Matsuo, Wei-Shau Hu, Vinay K. Pathak

**Affiliations:** 1 Viral Mutation Section, HIV Dynamics and Replication Program, Center for Cancer Research, National Cancer Institute, Frederick, MD 21702 USA; 2 Basic Research Laboratory, Leidos Biomedical Research, Inc., Frederick National Laboratory, Frederick, MD USA; 3 Viral Recombination Section, HIV Dynamics and Replication Program, Center for Cancer Research, National Cancer Institute, Frederick, MD USA

**Keywords:** APOBEC3G, CBFβ, HIV-1, Proteasomal degradation, Vif, Restriction factor

## Abstract

**Background:**

HIV-1 Vif interacts with the cellular core-binding factor β (CBFβ) and counteracts the protective roles of certain human APOBEC3 (A3) proteins by targeting them for proteasomal degradation. Previous studies have identified some amino acids important for Vif–CBFβ interactions, and recently a co-crystal structure of a pentameric complex of HIV-1 Vif, CBFβ, Cul5, EloB, and EloC was resolved. However, a comprehensive analysis of Vif–CBFβ interactions that are important for Vif function has not been performed.

**Results:**

Here, we carried out double-alanine scanning mutagenesis of the first 60 amino acids of Vif and determined their effects on interaction with CBFβ and their ability to induce A3G degradation as well as rescue HIV-1 replication in the presence of A3G. We found that multiple Vif residues are involved in the extensive N-terminal Vif–CBFβ interaction and that the ^5^WQVMIVW^11^ region of Vif is the major determinant. A minimum of three alanine substitutions are required to completely abrogate the Vif–CBFβ interaction and Vif’s ability to rescue HIV-1 infectivity in the presence of A3G. Mutational analysis of CBFβ revealed that F68 and I55 residues are important and participate in a tripartite hydrophobic interaction with W5 of Vif to maintain a stable and functional Vif–CBFβ complex. We also determined that CBFβ amino acids ^73^WQGEQR^78^, which are not resolved in the structure of the pentameric complex, are not involved in interaction with HIV-1 Vif.

**Conclusions:**

Our results provide detailed insight into the Vif–CBFβ interactions that are critical for Vif function and may contribute to the rational design of HIV-1 inhibitors that block Vif-mediated degradation of A3 proteins.

**Electronic supplementary material:**

The online version of this article (doi:10.1186/s12977-017-0346-5) contains supplementary material, which is available to authorized users.

## Background

HIV-1 and other lentiviruses encode the accessory protein Vif that counteracts the antiviral activities of APOBEC3 (A3) proteins and is required for infection and propagation in primary CD4^+^ T cells and in non-permissive T cell lines (for recent reviews see Refs. [1, 2]). Vif neutralizes the inhibitory activities of A3 proteins by targeting them for polyubiquitination and proteasomal degradation by hijacking an E3 ubiquitin ligase complex [3].

Jager et al. [4] and Zhang et al. [5] recently reported that in addition to binding to cullin 5 (Cul5) and elongin B/C (EloB/C), Vif binds to the cellular core-binding factor β (CBFβ), and the Vif–CBFβ interaction is essential for inducing efficient degradation of A3 proteins, which inhibit HIV-1 replication in non-permissive cell types. CBFβ is an evolutionarily conserved non-DNA binding component of the mammalian runt-related transcription factors (RUNX 1-3), which are critical in hematopoiesis, T cell differentiation, and skeletal development [6, 7]. CBFβ is an allosteric regulator of RUNX proteins and has previously been reported to stabilize RUNX1 by preventing its ubiquitin-mediated degradation [8]. Recently, CBFβ was proposed to play a critical role in stabilizing the intrinsically unstructured HIV-1 Vif and promoting formation of a well-ordered substrate receptor by facilitating local folding of the N-terminal Vif region [4, 5, 9–11].

Several reports have established that HIV-1 Vif interacts with CBFβ and have shown that CBFβ depletion hampers virus replication in cells expressing A3 proteins primarily by interfering with Vif’s ability to induce degradation of the A3 proteins [4, 5, 9]. HIV-1 Vif associates with CBFβ in human cells and recombinant CBFβ enhances Vif’s solubility, stability, and association with an E3 ubiquitin ligase complex [9, 10, 12–14]. Initially, the interaction interface was mapped to residues 15–126 of CBFβ [9, 15]; CBFβ amino acid F68 was then reported to be critical for stable HIV-1 Vif–CBFβ interaction [14]. Subsequently, an X-ray crystal structure of five pentameric complexes, each containing CBFβ–Vif–EloB/C-Cul5, was determined [10]. In the pentameric complex, HIV-1 Vif is tightly associated with CBFβ and interacts with both EloC and Cul5.

Determining the biological relevance of the critical residues of Vif that govern the specificity of the Vif–CBFβ interaction can facilitate our understanding of this important host–virus interaction. Additionally, elucidating this interaction at the molecular level can potentially assist in the design of inhibitors that block Vif–CBFβ binding and thereby interfere with Vif function. Previous mutational analyses identified several residues of Vif that were important for its interaction with CBFβ [10, 16–18]. Of particular relevance to this work, Fribourgh et al. [16] reported that deletion of the first 13 amino acids of Vif abolished the interaction of recombinant Vif with CBFβ, consistent with the crystal structure data, which showed that there are extensive interactions between the N-terminal hydrophobic anti-parallel β-strands of HIV-1 Vif and CBFβ [10].

To determine the functional importance of these extensive N-terminal HIV-1 Vif–CBFβ interactions, we analyzed double-alanine mutants of the first 60 amino acids of HIV-1 Vif and determined the effects of the mutations on CBFβ binding by co-immunoprecipitation. We found that ^5^WQVMIVW^11^ region of Vif is important for the Vif–CBFβ interaction, of which W5 is the most critical determinant. We also found that H27–H28 and D37–W38 influence CBFβ binding eventhough they are not at the CBFβ–Vif interface in the pentameric complex, suggesting that they indirectly affect the interaction with CBFβ. We also observed that CBFβ amino acids ^73^WQGEQR^78^, which are in close proximity to residues E45–S52 of Vif and are not resolved in the pentameric complex crystal structure, do not contribute to the Vif–CBFβ interaction. Finally, consistent with the crystal structure, our mutational analysis indicated that CBFβ amino acids I55 and F68 form a tripartite interaction with W5 of Vif that is critical for Vif function.

## Results

### Identification of HIV-1 Vif determinants that are important for interaction with CBFβ

**Fig. 1:**
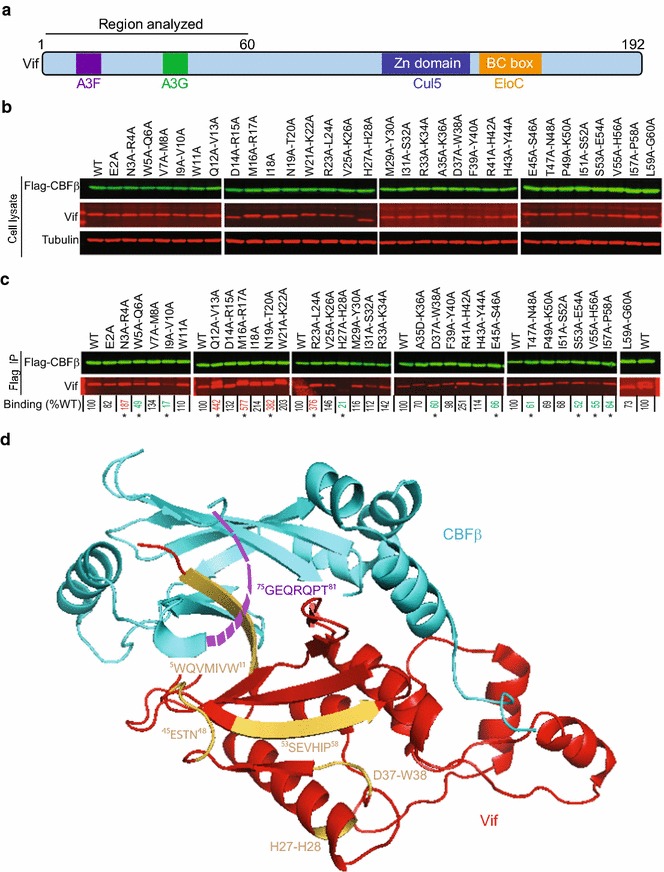
Identification of Vif determinants that are critical for binding to CBFβ. **a** A schematic of Vif structure and its interaction domains with cellular proteins APOBEC3F (A3F), APOBEC3G (A3G), cullin 5 (Cul5) and elongin C (EloC). Double alanine mutants of the first 60 amino acids of Vif were analyzed. **b** Immunoblots of cell lysates to detect Flag-CBFβ, Vif, and tubulin (used as loading control). **c** Immunoblots of WT and mutant Vifs that co-immunoprecipitated with Flag-CBFβ. The average binding efficiency of Vif mutants to CBFβ from five independent transfection experiments is shown below each lane relative to WT Vif (set to 100%). Statistical significance was determined using *t* test; *P < 0.05. The mutants with significantly reduced Vif–CBFβ binding are shown in *green*; mutants with increased Vif–CBFβ binding are shown in *red*. The *upper bands* of the Vif IP blots of Q12A-V13A and WT (the last panel) are most likely the light chains of the anti-Flag antibody used for immunoprecipitation. **d** A *ribbon diagram* of HIV-1 Vif; the highlighted residues ^5^WQVMIVW^11^, H27–H28, D37–W38, ^45^ESTN^48^, and ^53^SEVHIP^58^ decrease CBFβ binding efficiency as shown in (**c**). Vif ^45^ESTNPKIS^52^ residues are in a loop and beginning of a β-strand; these Vif amino acids are near a loop of CBFβ that was not resolved in the structure (^75^GEQRQPT^81^, *purple dashed line*). Vif is shown in *red* and CBFβ is shown in *cyan* (PDB:4N9F)

To identify HIV-1 Vif determinants that are essential for interaction with CBFβ, we used a previously described panel of single or double-alanine substitution mutants of the first 60 amino acids of Vif [19]. Our alanine-scanning mutagenesis screen focused primarily on identifying residues that abrogated the interaction between Vif and CBFβ as determined by co-immunoprecipitation (co-IP) assays (Fig. 1). We co-transfected 293T cells with plasmids encoding Flag-CBFβ and untagged Vif mutants, performed co-IP assays using anti-Flag antibody and lysates of the co-transfected cells and estimated the amounts of Vif proteins that co-immunoprecipitated with Flag-CBFβ using quantitative western blotting analysis (Fig. 1). The levels of the different Vif mutant proteins were comparable when they were expressed in the presence of Flag-CBFβ (Fig. 1a, b). The efficiency of CBFβ binding to wild-type (WT) Vif was set to 100%, and used to compare the binding efficiencies of the Vif mutants (Fig. 1b, c). The average binding efficiencies from five independent experiments are shown below for each mutant. Notably, of the mutants examined, W5A–Q6A, I9A–V10A, H27A–H28A, and D37A–W38A significantly reduced Vif’s ability to bind to CBFβ compared to WT Vif (Fig. 1b; values indicated in green). The H27A–H28A and D37A–W38A amino acids are not at the Vif–CBFβ interface. The solvent accessible surface area (SASA; determined by using getarea program available at http://curie.utmb.edu/getarea.html) for H28 is only 4.86 Å^2^, and W38 is not surface exposed (SASA is 0.0 Å^2^), suggesting that these amino acids are buried in the Vif protein, and substitution of these amino acids with alanines may have disrupted the overall structure of the α-domain of Vif. The locations of these amino acids are shown in Fig. 1d. In addition, mutations in the E45–P58 strand–helix–loop structure of Vif also exhibited partial defects in binding to CBFβ; mutant E45A–S46A, T47–N48A, S53A–E54A, V55A–H56A, and I57A–P58A showed significantly lower binding than WT Vif (Fig. 1c, d; values indicated in green). These results led to the identification of several amino acids in the N-terminal Vif region that are critically important for binding to CBFβ.

The pentameric structure [10] indicated that the Vif amino acids from W5–W11 are in close proximity to CBFβ amino acids N63–F68 and I55; each of the Vif amino acids is within 3.46–5.99 angstroms (Å) of the CBFβ amino acids, suggesting that any or all of these Vif–CBFβ interactions could be critical for Vif function. The importance of the W5–W11 amino acids of Vif is supported by a previous study [16] indicating that deleting the first 13 amino acids of Vif abolishes the interaction between Vif and CBFβ. Our results indicating that the double mutants W5A–Q6A and I9A–V10A disrupt the interaction with CBFβ, but the V7A–M8A and W11A mutants did not, indicated that some of these amino acids are critical for the interaction while others are less important. Surprisingly, our results showed that the H27A–H28A and D37A–W38A mutants severely hampered the interaction between Vif and CBFβ in our binding assay; these amino acids are 11–15 Å away from the nearest CBFβ amino acid (R151) in the pentameric structure and are not at the interaction interface (PDB:4N9F) [10]. Analysis of the structure of Vif [10] suggested that W38, I57, I107, Y111, and F112 are involved in an extensive hydrophobic interaction that maintains the structural organization of the α-domain of HIV-1 Vif (Additional file 1: Supplementary Fig. S1), implying that they indirectly affect the interaction with CBFβ.

Our observations that the double mutants W5A–Q6A and I9A–V10A disrupt the interaction with CBFβ in cells provide functional insights into the recently reported structural information of the pentameric complex [10], which showed that amino acids ^5^WQVMIVW^11^ interact with CBFβ. We also found that amino acids E45–P58 of Vif, which forms a flexible loop [10], influenced the Vif–CBFβ interaction. Amino acids E45–S52 are in a position to potentially form direct interactions with amino acids W73–R78 of CBFβ. However, this region of CBFβ was not resolved in the pentameric complex [10]; thus, a potential direct interaction between these amino acids of Vif and CBFβ could not be verified or excluded based on the crystal structure. Finally, we also observed that N3A–R4A, Q12A–V13A, M16A–R17A, N19A–T20A, and R23A–L24A mutants exhibited a significantly higher efficiency of binding to CBFβ compared to WT Vif (Fig. 1b; values indicated in red); the potential biological significance of increased binding to CBFβ by these mutants was not further examined in these studies.

### Effects of CBFβ-binding mutations on Vif’s ability to induce A3G degradation and rescue HIV-1 infectivity

**Fig. 2:**
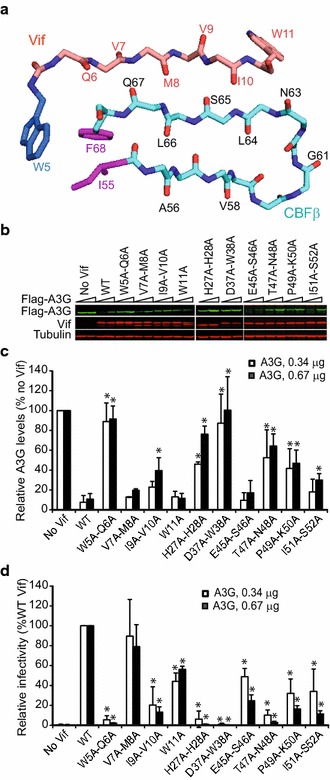
Effect of selected double-alanine mutations in Vif on A3G degradation and HIV-1 infectivity. **a** Stick diagram of amino acids at the Vif–CBFβ interface. ^5^WQVMVIW^11^ amino acids of Vif and amino acids 55–68 of CBFβ, and the interaction between W5 of Vif with I55 and F68 of CBFβ are shown. **b** Representative immunoblots of Flag-A3G, Vif, and tubulin (loading control). Degradation of Flag-A3G was determined by co-transfection of either 0.34 or 0.67 μg of Flag-A3G expression plasmid. **c** Quantitation of Vif-induced A3G degradation relative to the no Vif control (which was set to 100%) is shown. *Bar graphs* represent mean and standard deviations of two or more independent transfection experiments and statistical significance was determined using *t* test; *P < 0.05. **d** Infectivity of viruses in the presence of different Vif mutants. Data (mean ± SDs from two or more independent experiments done in triplicates) are plotted relative to viruses produced in the presence of WT Vif (set to 100%). Statistical significance was determined using *t* test; *P < 0.05

Next, we examined whether the Vif mutants that exhibited reduced binding to CBFβ showed a defect in their ability to induce A3G degradation and inhibit HIV-1 replication. We co-transfected 293T cells with HDV-GFP, VSV-G, and WT Vif (or Vif mutants) in combination with different amounts of A3G expression plasmids and examined the ability of the Vif mutants to mediate A3G degradation and rescue infectivity of HIV-1 (Fig. 2). In addition to the Vif mutants that were defective in CBFβ binding, we included V7A–M8A and W11A because they can potentially contribute to the extensive hydrophobic interaction between Vif and CBFβ anti-parallel β-strands. In addition, we included double mutants P49A–K50A and I51–S52A, because of their proximity to other double mutants that showed significant defects in CBFβ binding. Vif mutants W5A–Q6A, H27A–H28A, D37A–W38A, T47A–N48A, P49A–K50A, and I51A–S52A exhibited significant defects in their ability to mediate A3G degradation compared to WT Vif (Fig. 2b, c). Mutants V7A–M8A, I9A–V10A, W11A, and E45A–S46A induced A3G degradation as well as WT Vif. We next compared the infectivity of viruses produced in the presence of these Vif mutants to WT Vif (set to 100%); infectivity was significantly reduced for all of the mutants except V7A–M8A (Fig. 2d). Although the steady-state levels of A3G in the presence of W11A and E45A–S46A Vif mutants were similar to the level of A3G in the presence of WT Vif (Fig. 2b, c), there was a two-fold reduction in the infectivity of viruses produced in the presence of these Vif mutants (Fig. 2d). These results indicate that when A3G levels are low and barely detectable on western blots, virus infectivity is a more sensitive measure of the levels of A3G remaining than western blotting. Taken together, these results strongly indicate that Vif residues W5–Q6, I9–V10, H27–H28, D37–W38, and the loop region E45–S52 are critical for Vif’s ability to bind to CBFβ, induce A3G degradation, and rescue infectivity.

### Effect of reducing the level of CBFβ on the ability of Vif mutants to degrade A3G and rescue HIV-1 infectivity

**Fig. 3:**
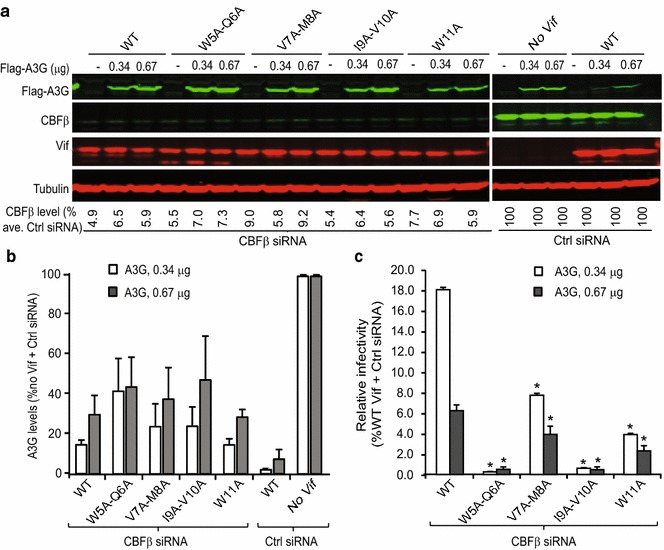
Effect of combination of double-alanine Vif mutants in CBFβ-depleted cells on Vif activity. **a** Representative western blots showing levels of A3G, Vif, CBFβ, and tubulin in cells co-transfected with Flag-A3G, Vif variants, and CBFβ-specific siRNA or control siRNA. Lysates of virus producer cells were collected 48 h post-transfection and analyzed by western blotting. Tubulin was used as a loading control for quantitation A3G levels. Quantitation of CBFβ knockdown efficiency from two independent experiments is depicted below each lane. **b** Quantitation of Vif-induced A3G degradation relative to the no Vif control (control siRNA; set to 100%) from two independent experiments. **c** Infectivity of viruses produced in experiments shown in (**a**) is plotted relative to virus produced in the presence of WT Vif and control siRNA (set to 100%; not shown). Infectivity was determined by measuring luciferase activity in TZM-bl cells. Statistical significance was determined using *t* test; *P < 0.05

Vif amino acids ^5^WQVMIVW^11^ form a β-strand that is at the CBFβ interface [10]; however, our alanine-scanning mutagenesis suggested that only W5A–Q6A and I9A–V10A mutants significantly reduced the interaction with CBFβ. We hypothesized that the other amino acids in the ^5^WQVMIVW^11^ motif contribute to CBFβ binding, but because there are high levels of CBFβ in 293T cells, modest reductions in CBFβ binding would not be observed in our co-IP assays. We therefore sought to assess whether reducing CBFβ levels in cells by siRNA knockdown would more readily reveal minor interactions that contribute to Vif–CBFβ binding. We first determined the effect of CBFβ depletion on A3G degradation by transfecting either a CBFβ-specific siRNA or a control siRNA (Fig. 3a, b). The results showed that the CBFβ-specific siRNA-mediated knockdown reduced CBFβ protein to 4.9–9.0% of the levels in the presence of control siRNA. We also determined the combined effect of CBFβ knockdown and Vif mutants on infectivity of viruses produced in the presence of 0.34 μg or 0.67 μg of A3G plasmid (Fig. 3c). The results showed that virus produced in cells with reduced levels of CBFβ and WT Vif resulted in further reduced infectivity compared to the infectivity in the presence of control siRNA (18 and 6% in the presence of 0.34 and 0.67 μg of A3G, respectively). When CBFβ was depleted and the V7A–M8A and W11A mutants were expressed, we observed a more significant defect in restoring virus infectivity (8 and 4% of the WT Vif in the presence of control siRNA, respectively). Thus, in the presence of normal levels of CBFβ, the V7A–M8A and W11A mutants either showed no defect or a modest twofold defect in rescuing virus infectivity compared to WT Vif (Fig. 3c), but in CBFβ-depleted cells, the same mutants showed significant defects in rescuing virus infectivity (approximately threefold and 4.5-fold, respectively). Taken together, these data indicate that the V7A–M8A and W11 residues do make a minor contribution to CBFβ binding, but their contribution to CBFβ binding is dependent on the CBFβ protein expression level.

### Effect of Vif quadruple– and triple–alanine substitution mutations ^5^WQVM^8^ > 4A and ^9^IVW^11^ > 3A on interaction with CBFβ and A3G degradation

**Fig. 4:**
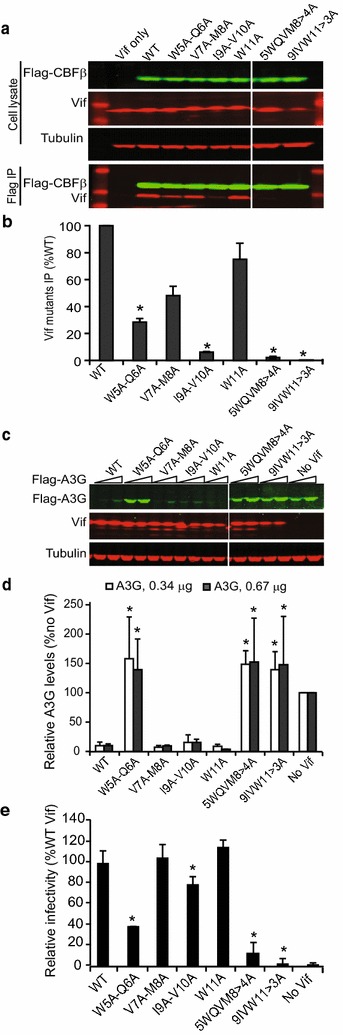
Effect of quadruple– and triple–alanine substitution mutants of Vif on CBFβ interaction and A3G degradation. Comparison of selected double, quadruple– (^5^WQVM^8^ > 4A), and triple– (^9^IVW^11^ > 3A) alanine substitution mutants of Vif with respect to CBFβ binding, A3G degradation, and ability to rescue virus infectivity. **a** Representative immunoblots of cell lysates (*upper three panels*) and co-IP (*lower panel*) probed for Flag-CBFβ, Vif, and tubulin (loading control) are shown. **b** Average efficiency with which Vif mutants bind to CBFβ from two independent transfection experiments relative to WT Vif (set to 100%). Statistical significance was determined using *t* test; *P < 0.05. **c** A representative immunoblot showing the effect of Vif mutants on A3G degradation. **d** Quantitation of relative A3G levels compared to the no Vif control (set to 100%).* Error bars* indicate standard deviations from four independent experiments. Statistical significance was determined using *t* test; *P < 0.05. **e** Infectivity of viruses produced in 293T cells in the presence of Flag-A3G and the indicated Vif mutants; mean ± SDs are for virus stocks obtained from two independent transfection experiments; virus infectivity of each virus stock was an average of three infections. All comparisons were based on viruses produced in the presence of WT Vif (set to 100%). Statistical significance was determined using *t* test; *P < 0.05

The double-alanine substitution mutations in ^5^WQVMIVW^11^ binding motif of HIV-1 Vif did not completely abolish CBFβ binding, suggesting that multiple residues are involved in the interaction between Vif and CBFβ [10]. Moreover, single-alanine substitution mutagenesis in the ^5^WQVMIVW^11^ motif of Vif revealed that only the W5A mutant significantly affected Vif’s activity against A3G and failed to rescue infectivity (Additional file 2: Supplementary Fig. S2). Because single amino acid substitutions ^6^QVMIVW^11^ did not have a significant effect on Vif–CBFβ binding, we sought to determine whether combining multiple substitutions in this region would influence Vif interaction with CBFβ. We constructed two additional mutants in which four or three Vif amino acids were substituted with alanines to generate ^5^WQVM^8^ > A4 and ^9^IVW^11^ > A3 Vif mutants. Consistent with our hypothesis, both ^5^WQVM^8^ > A4 and ^9^IVW^11^ > A3 mutants almost completely eliminated Vif binding to CBFβ with binding efficiencies of 2 and 0.3%, respectively (Fig. 4a, b).

Next, we determined the effects of the quadruple– and triple–alanine substitution mutations in Vif on A3G degradation and infectivity (Fig. 4c–e). We found that both ^5^WQVM^8^ > A4 and ^9^IVW^11^ > A3 mutants failed to mediate A3G degradation (Fig. 4c, d) and rescue infectivity when viruses were produced in cells that co-expressed A3G (Fig. 4e). These data suggest that the N-terminal interaction between HIV-1 Vif and CBFβ is stabilized by hydrophobic interactions and at least three alanine substitutions are required to abolish the interaction between the anti-parallel β-strands of Vif and CBFβ [10].

### ^73^WQGEQR^78^ residues of CBFβ are not important for stable Vif–CBFβ interaction

**Fig. 5:**
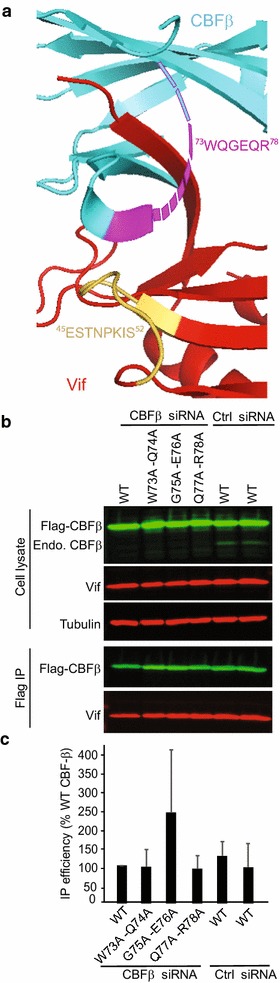
Effect of mutations in the ^73^WQGEQR^78^ region of CBFβ on Vif–CBFβ interaction. **a** A ribbon diagram of HIV-1 Vif and CBFβ; Vif residues ^45^ESTNPKIS^52^ are shown in *orange* and CBFβ residues ^73^WQGEQR^78^ are shown in *purple*. The *dashed line* indicates loop region of CBFβ that was not resolved in the structure. Vif is shown in *red* and CBFβ is shown in *cyan* (PDB: 4N9F). **b** After transfection of 293T cells with CBFβ-specific or control siRNA, the cells were co-transfected with WT CBFβ or double-alanine substitution mutants of CBFβ and WT Vif. Immunoblots of cell lysates (*upper panel*) and co-IP samples (*lower panel*) probed for Flag-CBFβ, endogenous CBFβ, Vif, and tubulin (loading control) are shown. **c** The *bar graphs* represent the average immunoprecipitation efficiency of the different CBFβ mutants to WT Vif relative to WT CBFβ (set to 100%). None of the mutations had a measurable effect on CBFβ levels or on Vif–CBFβ binding

After identifying the Vif determinants that interact with CBFβ, we sought to determine the role of CBFβ determinants that are in close proximity to Vif in the co-crystal structure and may contribute to the Vif–CBFβ binding [10]. We first investigated whether ^73^WQGEQRQTPS^82^ residues in a flexible strand-loop region of CBFβ are important for interaction with Vif residues E45–S52 (Fig. 5a). Unfortunately, the CBFβ residues ^75^GEQRQTP^81^ are not resolved in the pentameric crystal structure [10] (Fig. 5a; dashed line). After analysis of the pentameric structure, we decided to investigate ^73^WQGEQR^78^ residues, which are potentially in close proximity to the Vif residues E45–S52.

We generated CBFβ mutants W73A–Q74A, G75A–E76A, and Q77A–R78A and determined their ability to interact with Vif (Fig. 5b, c). To examine the effects of these CBFβ double mutations on Vif binding, we first knocked down the endogenous CBFβ in 293T cells using an siRNA that binds to the 3′ untranslated region of the CBFβ mRNA; the efficiency of knockdown was >90% (Fig. 5b). We then expressed wild-type Flag-CBFβ or its mutants and determined the effects of the mutations on steady-state levels of Flag-A3G. We found that none of these CBFβ double mutants had a measurable effect on the interaction between CBFβ and Vif compared to wild-type CBFβ (Fig. 5b, c), suggesting that the ^73^WQGEQR^78^ residues are not required for the Vif–CBFβ interaction.

### A tripartite interaction between I55 and F68 of CBFβ and W5 of Vif is critical for stable Vif–CBFβ binding

**Fig. 6:**
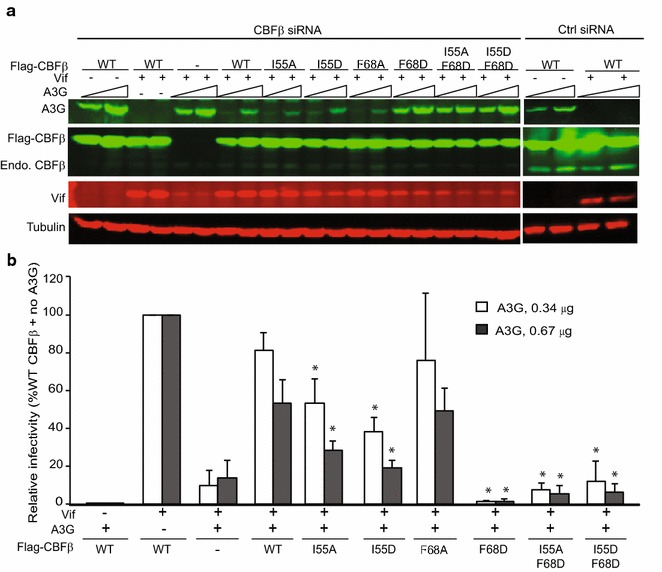
Identification of CBFβ mutants that are defective in Vif binding and restoring infectivity in the presence of A3G. **a** Representative western blots of 293T cells co-transfected with CBFβ siRNA or control siRNA, along with Flag-CBFβ, WT Vif, and 0.34 or 0.67 μg of Flag-A3G. The blots show Flag-A3G, Flag-CBFβ, endogenous CBFβ, WT Vif, and tubulin (loading control). **b** Infectivity of viruses produced in experiments shown in (**a**) as determined by measuring luciferase activity in TZM-bl cells. The *bar graphs* represent relative infectivity compared to no A3G samples (set to 100%). All data represent mean ± SDs from two independent transfection experiments; virus infectivity for each virus stock was an average of three infections. Statistical significance was determined using *t* test; *P < 0.05

The major interaction between CBFβ and N-terminal Vif residues involves two anti-parallel β-strands. Based on the pentameric crystal structure, the distance between W5 and I55 is 3.8 Å, W5 and F68 is 4.4 Å, and I55 and F68 is 4.4 Å. We hypothesized that W5 of Vif and I55 and F68 residues of CBFβ form a tripartite interaction that is critical for Vif–CBFβ binding. To confirm that this interaction is important in cells, we mutated the CBFβ residues I55 and F68 and measured the impact that these mutations had in co-IP assays in which the endogenous CBFβ was depleted by siRNA. Hultquist et al. previously showed that the F68D mutant of CBFβ differentially abrogated the interaction between CBFβ and Vif while preserving CBFβ’s ability to bind to its cellular interaction partner RUNX1 [14]. In addition to the F68D mutant, we generated CBFβ mutants I55A, I55D, F68A, I55A–F68D, and I55D–F68D and examined their ability to interact with Vif (Fig. 6). To examine the effects of the mutations in CBFβ on Vif binding, we first knocked down the endogenous CBFβ in 293T cells using siRNAs that bind to the 3′ untranslated region of the CBFβ; the efficiency of knockdown was >90%. We then expressed Flag-tagged wild-type CBFβ or its mutants and determined the effect of the mutations on steady-state levels of Flag-A3G (Fig. 6a, b). The results showed that, compared to CBFβ-WT, the I55A and I55D mutants were less efficient at restoring virus infectivity in the presence of 0.34 and 0.67 μg of A3G. The F68A substitution did not influence Vif–CBFβ interaction in this assay and could restore virus infectivity to the same extent as CBFβ–WT. In agreement with previously reported results [14], the F68D mutant was unable to rescue virus infectivity, as were both the I55A–F68D and I55D–F68D double mutants (Fig. 6a, b). These results indicated that in addition to F68, I55 affects Vif–CBFβ interaction.

**Fig. 7:**
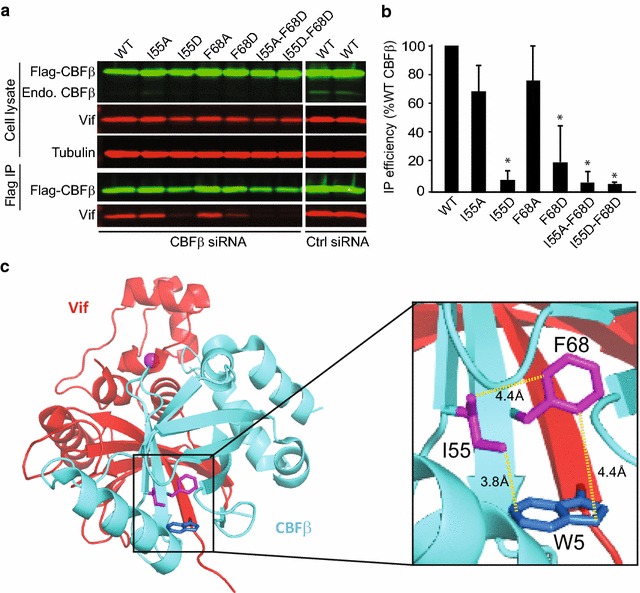
The tripartite interaction between I55 and F68 of CBFβ and W5 of Vif is sufficient to stabilize Vif–CBFβ interaction. **a** After treatment of 293T cells with CBFβ-specific siRNA, the cells were co-transfected with WT Flag-CBFβ or substitution mutants of Flag-CBFβ and WT Vif. The cells were treated with MG132 (5 μM) for 16-h before cell lysates were harvested. Immunoblots of cell lysates (*upper three panels*) and co-IP samples (*lower two panels*) probed for Flag-CBFβ, endogenous CBFβ, Vif, and tubulin (loading control) are shown. **b** Average immunoprecipitation efficiency of WT Vif with CBFβ mutants relative to WT CBFβ (set to 100%). Statistical significance was determined using *t* test; *P < 0.05. **c** The model depicts Vif (*red*) and CBFβ (*cyan*) adapted from PDB: 4N9F. The tripartite hydrophobic interaction of I55 (*magenta*) and F68 (*magenta*) of CBFβ and W5 (*blue*) of Vif are *highlighted in black frame* and a close-up of the highlighted residues is shown with the approximated distance between them in Å

Finally, we examined the ability of the CBFβ mutants to bind to HIV-1 Vif (Fig. 7a, b). Both I55A–F68D and I55D–F68D CBFβ double mutants almost completely failed to bind to Vif, reducing binding efficiency to ~8% of that observed for CBFβ-WT in the co-IP assay (Fig. 7b). Individually, the I55D and F68D mutants significantly reduced Vif binding to ~15 and ~28% compared to that of CBFβ-WT, respectively (Fig. 7b), suggesting that disruption of the tripartite interaction almost completely inhibits the Vif–CBFβ interaction. As expected, substituting I55 with the less bulky hydrophobic residue (A55) had only a modest effect on Vif binding (Fig. 7a, b). Taken together, these results show that the tripartite interaction between I55 and F68 of CBFβ and W5 of Vif is critical for Vif’s ability to induce A3G degradation and restore virus infectivity (Fig. 7c).

## Discussion

The list of host proteins in the Vif interactome is expanding and among these are EloB/C, CUL5, and CBFβ, which are essential for counteracting the antiviral activities of A3 proteins by targeting them for proteasomal degradation [4, 5, 10, 20–22]. Thus, the Vif–CBFβ interaction is a potential target for development of antiviral agents that interfere with Vif’s ability to induce degradation of A3 proteins. The recently reported pentameric crystal structure of Vif–CBFβ–CUL5–EloB/C has provided valuable structural information about this interaction [10], and has indicated that the interface is stable primarily due to extensive hydrophobic interactions involving a large surface area (4797 Å^2^). Although the extensive nature of this protein–protein interaction suggests that small molecules are unlikely to disrupt the Vif–CBFβ interaction, it is possible that specific determinants of this interaction are essential for Vif function, and such functionally relevant determinants could provide targets for antiviral drug development. In these studies, we sought to determine which Vif–CBFβ interactions are essential for Vif function by performing alanine scanning mutagenesis of the first 60 amino acids of Vif. Our results show that Vif residues ^5^WQVMIVW^11^ play a critical role in binding to CBFβ; specifically, our results indicate that W5 of Vif forms a critical tripartite interaction with I55 and F68 of CBFβ. Substitution of any one of these three residues almost completely abolished the binding of Vif to CBFβ, as determined in Co-IP assays. Mutation of W5 of Vif abolished Vif’s ability to induce degradation of A3G, and mutants of CBFβ with substitutions at the I55 and F68 positions failed to rescue Vif’s function when the endogenous CBFβ was depleted by siRNA knockdown. Our results confirm a previous study indicating that F68D mutation of CBFβ abrogated Vif–CBFβ interaction without affecting the formation of the CBFβ-RUNX1 heterodimer [14], and other studies [16, 18] which indicated that Vif amino acids ^5^WQVMIVW^11^ are important for CBFβ binding. Unlike the previous studies, which performed deletion and substitution mutation analysis in the absence of a structure, we performed a rational, structure-based systematic analysis to map the amino acid determinants that are functionally critical for Vif–CBFβ interaction. Importantly, our results indicate that the W5 of the ^5^WQVMIVW^11^ is critical for CBFβ binding and participates in a tripartite interaction with CBFβ residues I55 and F68, while other amino acids make minor contributions to CBFβ binding. We also showed that residues of a disordered loop of CBFβ (amino acids 73–78 of loop 3), which are not modeled in the crystal structure due to poorly defined electron density, are dispensable for interactions with Vif.

We found that the other amino acids in the ^5^WQVMIVW^11^ motif did contribute to the interaction with CBFβ, but 3 or 4 alanine substitution mutations were required to completely abolish CBFβ binding. The ^5^WQVMIVW^11^ forms a β strand that makes β barrel-like interactions with the β2 and β3 strands of CBFβ. Since backbone hydrogen bonds, and not side-chain bonds, are critical for formation of β barrels, it is possible that multiple alanine substitutions are required to alter the secondary structure of this motif, resulting in loss of interaction with CBFβ.

Our results indicated that Vif amino acids E45–S52 played a role in Vif–CBFβ binding. The exact mechanism by which these Vif residues contribute to the interaction with CBFβ is not clear. We mutated CBFβ residues W73–R78, which are not resolved in the pentameric structure [10] and are part of the previously reported CBFβ deletion mutant (amino acids 69–90) that abrogated its binding to HIV-1 Vif [5]. Our double-alanine substitution study showed that these CBFβ residues did not significantly contribute to Vif binding. Our results likely differ because the previous study analyzed a larger deletion compared to the region analyzed in our study (amino acids 69–90 vs. 75–81). The deletion most likely affected the overall structure of CBFβ, which resulted in loss of Vif binding; the double-alanine substitution mutants analyzed in our study did not have an effect on the structure of CBFβ and did not affect Vif binding. Thus, Vif residues E45–S52 do not compose a bona fide CBFβ interaction site; it is possible that substitution mutations of Vif residues E45–S52 may have indirectly affected the structure of Vif, resulting in a weaker interaction between W5 and I55 or F68. Although it was not discussed previously, we noted a potential electrostatic interaction between CBFβ E54 and Vif K50. Interestingly, CBFβ E54 is adjacent to I55, which is involved in the tripartite interaction; disruption of a potential electrostatic interaction between Vif K50 and CBFβ E54 may have indirectly influenced the I55 residue position, leading to a weaker binding to Vif.

The results of these studies indicate that Vif–CBFβ and Vif-APOBEC3 interaction surfaces do not overlap and are mutually exclusive. Mutations in the ^14^DRMR^17^ and ^40^YRHHY^44^ motifs, which were previously shown to be essential for degradation of A3F and A3G, respectively, did not have any effect on the Vif–CBFβ interaction. We also found that several Vif amino acids, including H27, H28, W38, and others are likely to be involved in a hydrophobic interaction that is essential for maintaining the structure of the α-domain of Vif and are not directly involved in interaction with CBFβ. These results are consistent with prior mutagenesis studies [17].

Results of prior mutagenesis studies [19, 23, 24] and the current study on the effect of residues H27, H28, W38, T47, N48, P49, K50, I57, P58, and F112 mutants of Vif on binding to CBFβ and APOBEC3 proteins can be interpreted and grouped into those that participate in an extensive hydrophobic interaction and those that maintain the structure of the α-domain of Vif. Previous studies found that the W21A mutant was unable to bind to CBFβ [5, 6, 17, 18], while our studies indicate that the W21A–K22A double mutant does not have any apparent defect in CBFβ binding. Examination of the pentameric crystal structure shows that the W21 residue of Vif is buried within the α-domain motif of Vif and does not interact with CBFβ; SASA for W21 is 6.49 Å^2^. Thus, the W21A mutation most likely results in a major structural change in the α-domain of Vif, resulting in loss of binding to CBFβ. Interestingly, our results suggest that the lysine residue at the 22 position is required for the structural changes induced by the W21A mutation.

The pentameric structure of Vif in complex with CBFβ suggests other potential interactions between Vif and CBFβ. Vif residues W89 is close to CBFβ residue F143, and Vif Y94 is in close proximity to CBFβ I102, E135, D136, and Q140. In addition, Vif C-terminal residues near F115 are in close proximity to CBFβ residue F153 and other residues between 151 and 156. Although these interactions likely stabilize Vif–CBFβ binding, they are not sufficient to retain Vif activity, since mutations at W5 of Vif, or I55 and/or F68 of CBFβ were sufficient to almost completely inhibit Vif’s ability to bind to CBFβ and induce A3G degradation. Although these interactions are not sufficient to support Vif function, additional studies are needed to determine whether other mutations at these sites can result in steric hindrance and interfere with Vif–CBFβ binding. Similarly, the functional significance of Vif–Cul5 and Vif–EloC interactions described in the pentameric structure should be determined to establish whether these interactions may be valuable targets for development of small molecule inhibitors.

## Conclusions

Our results provide detailed insight into the critical determinants of the interaction interface between the N-terminus of Vif and CBFβ and identified a tripartite interaction that plays a major role in Vif–CBFβ binding. Together with the available structural data on Vif–CBFβ–E3 ubiquitin ligase complex, these results further our understanding of the biology of HIV-1 Vif–CBFβ interaction and may aid in the development of novel therapeutics that target the Vif–CBFβ interaction and inhibit Vif-mediated degradation of A3 proteins.

## Methods

### Plasmids and cell lines

Expression plasmids of Flag-A3G, HIV-1 Vif and its N-terminal double-alanine substitution mutants, HIV-1 vector HDV-eGFP, and VSV-G expression plasmid phCMV-G were previously described [19, 25–31]. All Vif mutants were generated by site-directed mutagenesis using a QuickChange Lightening site-directed mutagenesis kit (Agilent Technologies) and verified by sequencing. The Flag-CBFβ expression plasmid was obtained from Addgene.

HeLa-derived reporter TZM-bl and 293T cell lines were maintained in DMEM (Corning Cellgro) supplemented with 10% fetal bovine serum (HyClone) and 1% penicillin–streptomycin (GIBCO).

### Co-immunoprecipitation assays and western blotting

Flag Co-IP assays were carried out as previously described [19, 25]. Briefly, 293T cells were seeded at 4 × 10^6^ cells per 10-cm dish and transfected the next day by the polyethylenimine PEI method [19, 32]. The following DNA amounts were used to transfect 293T cells: 4 μg Flag-CBFβ, 4 μg Vif, and 1.8 μg pGreen Lantern-1 (pGL) (GIBCO; control for transfections) expression plasmids. To maintain equivalent DNA amounts, pcDNA3.1 was used when needed. After 48 h, total cell lysates were harvested in 1 mL of lysis buffer and Co-IP was performed as previously described [19, 25]. To detect eluted complexes as well as the input cell lysates, western blotting was performed. CBFβ was detected using a rabbit anti-Flag polyclonal antibody (Sigma) at a 1:2000 dilution, Vif was detected using a mouse anti-Vif monoclonal antibody [Clone 319] (ab66643; Abcam) at a 1:2000 dilution, and tubulin as loading control was detected using mouse anti-tubulin antibody (Sigma) at a 1:20,000 dilution. Rabbit and mouse primary antibodies were detected using 1:5000 dilutions of an IRDye^®^ 800CW-labeled goat anti-rabbit secondary antibody (Licor) or an IRDye^®^ 680-labeled goat anti-mouse secondary antibody (Licor). Protein bands were visualized and quantified using an Odyssey^®^ Infrared Imaging System (Licor).

### A3G degradation assays

Human 293T cells were seeded at 8 × 10^5^ cells per well in six-well plates and transfections were carried out using the PEI method. To assay for Vif-mediated degradation of A3G, the following plasmids and amounts were used: 0.34 and 0.67 μg of pFlag-A3G, 2.5 μg of Vif, and 0.2 μg pGL (used as a positive control for transfection). To maintain equivalent DNA amount, pcDNA3.1 was added as needed. After 48 h, cell lysates were harvested and immunoblotting analyses were performed to detect steady-state expression levels of A3G in the absence or presence of Vif. Flag-A3G was detected using a rabbit anti-Flag polyclonal antibody (Sigma). HIV-1 Vif, endogenous CBFβ and tubulin were probed as described above.

### Virus production

To produce virus, 293T cells were seeded at 8 × 10^5^ cells per well in six-well plates, and transfected using the PEI method with the following plasmids and amounts: 1 μg of pHDV-eGFP, 0.25 μg of phCMV-G, 0.34 μg or 0.67 μg of pFlag-A3G, and 2.5 μg of Vif. To produce virus with or without CBFβ depletion, 293T cells were seeded at 2.5 × 10^5^ cells per well in six-well plates a day before transfection and the Lipofectamine2000 transfection method (Invitrogen) was used with the following plasmids and amounts: 1 μg of pHDV-eGFP, 0.25 μg of phCMV-G, 0.34 μg or 0.67 μg of pFlag-A3G, and 2.5 μg of Vif in combination with the specific siRNA as described below. After 48 h, the virus-containing supernatants were filtered through 0.45-μm filter and kept at −80 °C until use.

### Virus infectivity

TZM-bl cells were seeded in 96-well plates (4 × 10^3^ cells per well) and were infected the next day in triplicate with viruses normalized for p24 as determined by p24 ELISA (XpressBio). Forty-eight hours later, luciferase activity was determined by britelite™ plus kit (PerkinElmer) following the instructions of the manufacturer using a LUMIstar Galaxy luminometer or 1450 MicroBeta JET (PerkinElmer).

### CBFβ knockdown

For CBFβ knockdown experiments, 293T cells were seeded at 2.5 × 10^5^ cells per well in six-well plates a day before transfection. Small interfering RNA (siRNA)-mediated transient knockdown of CBFβ was performed using Silencer select CBFβ siRNA targeting coding region (Ambion; S2470) or siRNA-B07 targeting 3′-UTR region (Invitrogen; NM_022845.2_stealth_865), which was designed based on an shRNA described previously [4] and control siRNA at 25 nM using Lipofectamine RNAiMax reagent (Invitrogen) following the manufacturer instructions. Twenty-four hours later the cells were co-transfected with the respective siRNA (25 nM) and expression plasmids of Flag-A3G, Vif, VSV-G, and HDV-eGFP vector using Lipofectamine2000 reagent in order to produce virus for infectivity (see above for details of the amounts of plasmids used) and maintain efficient CBFβ knockdown. In some CBFβ overexpression rescue experiments, in addition to the above co-transfection cocktails of plasmids, we added either wild-type or different mutants (I55A, I55D, F68A, F68D, I55A-F68D, or I55D-F68D) of CBFβ expression plasmids and carried out infectivity and A3G degradation assays as described above. After 48 h, producer cell lysates and supernatants were collected for western blotting and infectivity assays, respectively.

## Additional files

**Additional file 1: Supplementary Fig. S1.** W38 and I57 are involved in maintaining the structural organization of the α-domain of Vif. A ribbon diagram of HIV-1 Vif highlighting residues W38, I57, I107, Y111, and F112 in *blue*. Vif is shown in *red* and CBFβ is shown in *cyan* (PDB: 4N9F). W38, I57, and I107 are not surface exposed (SASA 0.0 Å^2^); Y111 and F112 are only partially surface exposed (SASAs of 19.77 and 6.05 Å^2^, respectively).

**Additional file 2: Supplementary Fig. S2.** Effect of single-alanine substitution in the ^5^WQVMVIW^11^ motif of Vif on A3G degradation and infectivity. (**A**) Representative western blots showing levels of A3G, Vif, and tubulin in cells co-transfected with Flag-A3G and Vif variants. Lysates of virus producer cells were collected 48 h post-transfection and analyzed by western blotting. Tubulin was used as a loading control for quantitation A3G levels. (**B**) Quantitation of Vif-induced A3G degradation relative to the no Vif control set to 100% from two independent experiments. (**C**) Infectivity of viruses produced in experiments shown in panel A is plotted relative to virus produced in the presence of WT Vif set to 100%. Infectivity was determined by measuring luciferase activity in TZM-bl cells. Statistical significance was determined using *t* test; **P* < 0.05.
